# Exercise‐Induced Hypoalgesia Following Blood Flow Restriction in Rotator Cuff Repair Rehabilitation: A Randomized Crossover Clinical Trial

**DOI:** 10.1155/tsm2/4698970

**Published:** 2026-04-16

**Authors:** Felipe Ponce-Fuentes, Iván Cuyul-Vásquez, Joaquín Salazar-Méndez, Enrique Lluch, Luis Suso-Martí, Chad Cook, Filip Struyf, José Granell Casaña, Joaquín Calatayud

**Affiliations:** ^1^ Escuela de Kinesiología, Facultad de Medicina y Ciencias de la Salud, Universidad Mayor, Temuco, Chile, umayor.cl; ^2^ Faculty of Physiotherapy, University of Valencia, Valencia, Spain, uv.es; ^3^ Departamento de Procesos Terapéuticos, Facultad de Ciencias de la Salud, Universidad Católica de Temuco, Temuco, Chile, uctemuco.cl; ^4^ Facultad de Ciencias de la Salud, Universidad Arturo Prat, Sede Victoria, Chile, unap.cl; ^5^ Escuela de Ciencias Del Deporte y Actividad Física, Facultad de Salud, Universidad Santo Tomás, Talca, Chile, ust.cl; ^6^ Physiotherapy in Motion, Multi-Specialty Research Group (PTinMOTION), Department of Physiotherapy, University of Valencia, Valencia, Spain, uv.es; ^7^ Pain in Motion International Research Group, Vrije Universiteit Brussel, Brussels, Belgium, vub.ac.be; ^8^ Exercise Intervention for Health Research Group (EXINH-RG), Department of Physiotherapy, University of Valencia, Valencia, Spain, uv.es; ^9^ Department of Orthopaedics, Duke University School of Medicine, Duke University, Durham, North Carolina, USA, duke.edu; ^10^ Department of Rehabilitation Sciences and Physiotherapy, Faculty of Medicine and Health Sciences, University of Antwerp, Antwerp, Belgium, uantwerpen.be

**Keywords:** blood flow restriction training, exercise-induced hypoalgesia, isometric contraction exercise, rotator cuff repair, shoulder pain

## Abstract

**Objectives:**

To compare the acute effect on exercise‐induced hypoalgesia (EIH) between isometric exercise with blood flow restriction (BFR) and isometric exercise alone in adults undergoing arthroscopic RC repair and to determine the level of correlation between EIH and kinesiophobia, disability and quality of life.

**Design:**

Blinded randomized crossover clinical trial.

**Patients:**

Twenty‐three adults with degenerative rotator cuff (RC) repair.

**Intervention:**

Patients completed three shoulder isometric exercises. They were randomized to receive isometric exercises alone (control intervention) or isometric exercises with BFR (experimental intervention) in the first session and the alternate condition in the next session.

**Outcomes:**

Primary outcomes included pressure pain threshold (PPT) and conditioned pain modulation (CPM), assessed before, immediately after, and 10 min after isometric exercises with BFR and isometric exercises alone.

**Results:**

In the affected limb, BFR isometric exercises increased PPT immediately after exercise in upper trapezius (MD: 0.4 kg/cm^2^ [CI_95%_ 0.1 to 0.9]), and in the unaffected limb, BFR isometric exercises increased deltoid PPT immediately after exercise (MD: 0.4 kg/cm^2^ [CI_95%_ 0.1 to 0.7]). However, post hoc between‐group comparisons showed that no significant differences existed between BFR and control at any time point, as all CI_95%_ crossed zero and these changes did not exceed the minimum detectable change (≈1.1 kg/cm^2^). Besides, isometric exercise alone increased deltoid CPM immediately after in affected limb (MD: 30.1% [CI_95%_ 10.8 to 49.4]) and 10 min after exercise in unaffected limb (MD: 12.0% [CI_95%_ −0.4 to 24.4]). No significant changes were found in clinical pain intensity measured by the visual analog scale (VAS) at any time point. Statistically significant weak correlations were found between PPT with Western Ontario Rotator Cuff (WORC) index (*r* = −0.322 for sport, *r* = −0.331 for work, *r* = −0.302 for lifestyle; *p* < 0.05) and Shoulder Pain Disability Index (SPADI) (*r* = −0.309; *p* < 0.05), respectively.

**Conclusion:**

A single session of low‐intensity isometric exercises with BFR did not produce a clinically meaningful EIH response nor was it superior to isometric exercise alone in patients after RC repair. The observed statistical signals did not reach the minimum detectable change, and the inconsistent CPM shifts suggest that BFR does not provide an additional acute advantage for endogenous pain modulation in this early postoperative stage.

**Trial Registration:** ClinicalTrials.gov identifier: NCT06924112

## 1. Introduction

Rotator cuff (RC) tears are among the leading causes of shoulder pain and disability, accounting for approximately 20% of all shoulder injuries [1], with an increasing prevalence among aging populations [2]. Arthroscopic RC repair is a surgical procedure that has seen a 238% increase in the general population and 64% in people between 45 and 54 years of age [3]. Despite being characterized as a “minimally invasive” procedure, arthroscopic RC repair is associated with significant postoperative pain, especially in the first 2 weeks after surgery [4], which can compromise recovery time and rehabilitation goals.

The primary goal of the early stage of rehabilitation after RC repair is pain management, which is essential for improving the range of movement in the following stages [5]. There is currently controversy about the traditional postoperative rehabilitation protocol following arthroscopic RC repair, particularly for the early postoperative stage, which is characterized by a high irritability and low tolerance to movement [5, 6]. Moreover, aggressive early rehabilitation could compromise repair integrity [6] and adversely affect shoulder function in the long term [7]. Traditional postoperative protocols typically including early range of motion exercises are usually limited to passive mobilizations and pendulum exercises, with minimal shoulder active motion exercises [8]. This context limits exercise’s effect on pain control and movement tolerance in the acute stage of postoperative rehabilitation.

On the other hand, active exercise is a valuable strategy for pain management and has been shown to influence pain sensitivity in clinical musculoskeletal conditions [9] and settings [10]. This phenomenon, known as exercise‐induced hypoalgesia (EIH), refers to an acute reduction in pain sensitivity and/or perception of pain intensity to a noxious stimulus after exercise [11]. The physiological mechanisms of EIH involve enhanced descending endogenous pain inhibition systems, which secrete substances from opioid and endocannabinoid systems that have antinociceptive effects [12]. Isometric exercise has been shown to produce hypoalgesic effects in healthy adults [13]. However, the available evidence is not consistent with EIH after isometric exercise in people with shoulder pain [13]. Furthermore, it has been shown that increased EIH requires high intensity and longer duration of exercise, with higher load contractions that may also have a greater on the descending inhibitory system [14]. However, these recommendations may be counterproductive during the early stage of arthroscopic RC repair rehabilitation, as the tendon is in an early healing phase and shoulder contraction is barely tolerated [14]. In addition, EIH is modulated by biopsychosocial factors, such as kinesiophobia, that may attenuate endogenous inhibition [15]. Investigating the association between EIH, kinesiophobia, disability, and quality of life is crucial to identify if a patient’s psychological status attenuates the pain inhibitory response, especially during early postoperative recovery. In this context, alternative forms of traditional exercise such as low‐intensity blood flow restriction (BFR) training are emerging to maximize EIH in people with musculoskeletal conditions [16–18].

BFR exercise is an intervention that combines an external pressure system or cuff applied to a limb, partially restricting arterial blood flow and fully restricting venous blood flow, with low‐load resistance training [19]. The main advantage of BFR exercise is its ability to induce adaptations in skeletal muscle mass and strength with low‐intensity resistance exercises through various metabolic, hormonal, and neuromuscular pathways [20]. Recent studies have explored the effect of BFR exercise on EIH in individuals with musculoskeletal conditions [16–18]. This may be especially useful during the early stages of postoperative rehabilitation in patients undergoing RC repair, which is characterized by high local irritability and low exercise tolerance [21]. Although EIH with BFR exercise can have a significant clinical impact, evidence for its effects remains scarce on pain management in postoperative rehabilitation settings [16–18]. In this context, BFR exercise has shown promising short‐term results in various musculoskeletal cohorts; for instance, Constantinou et al. observed immediate pain reduction in patients with patellofemoral pain [22]. Previously, Korakakis et al. reported a significant hypoalgesic effect immediately after BFR exercise in people with anterior knee pain [16, 17]. Recently, Ogrezeanu et al. evaluated the effect of resistance training combined with BFR on EIH in patients with advanced knee osteoarthritis, indicating a significant increase in pain tolerance immediately and 10 min after BFR exercise [18]. While these findings in different clinical populations provide a rationale for investigating BFR‐induced hypoalgesia, their applicability to the specific context of postoperative RC repair requires direct investigation.

Although these data suggest a potential hypoalgesic effect for BFR exercise in people with musculoskeletal conditions, it remains unknown if these results generalize to patients undergoing RC repair rehabilitation. Specifically, no prior studies have examined EIH after BFR in postoperative RC patients, and there are a limited number of studies that explore the proximal effects of the BFR training due to anatomical constraints on cuff placement [23]. The primary aim of the study was to compare the acute effect on EIH between isometric exercise with BFR (experimental group) and isometric exercise alone (control group) in adults undergoing arthroscopic RC repair. The secondary aim was to examine correlations between EIH with kinesiophobia, shoulder pain–related disability and quality of life. We hypothesized that a single session of low‐intensity isometric exercise with BFR would elicit a greater EIH effect, characterized by an increase in pressure pain threshold (PPT) and enhanced conditioned pain modulation (CPM), compared to the same exercise without BFR in patients following RC repair.

## 2. Methods

This randomized controlled trial with a crossover design was approved by the Scientific Ethics Committee of the RedSalud Clinic of Chile (protocol number: P‐5.2025). The study adhered to the CONSORT extension for randomized crossover trials [24].

### 2.1. Study Design

**FIGURE 1:**
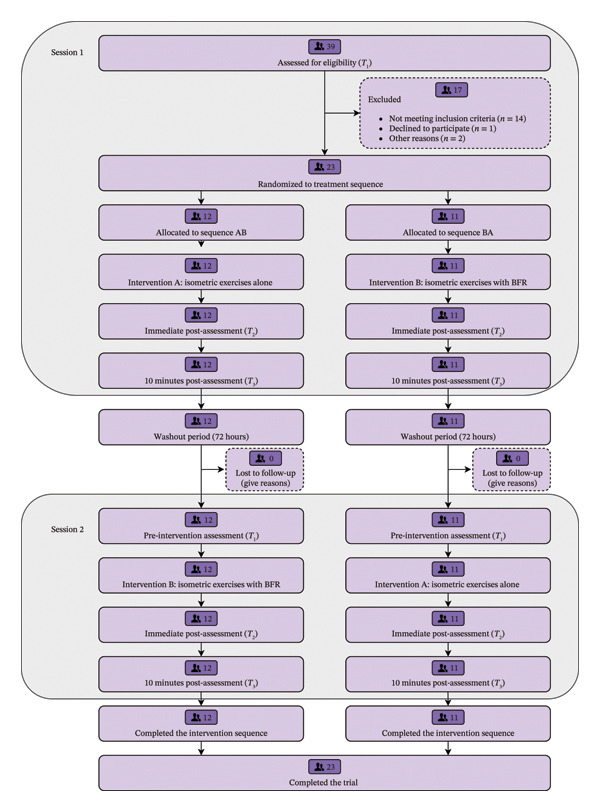
CONSORT flow diagram illustrating participant progression through the trial, including enrollment, allocation to intervention sequences (AB/BA), and analysis.

A randomized, controlled, assessor‐blind, 2 × 2 crossover (two periods and two interventions) trial was designed. For 1 week, each participant attended two sessions, separated by a 72‐h wash‐out period based on a previous similar study [25]. In each session, assessments were performed at baseline (*T*_1_), immediately after the intervention (*T*_2_), and 10 min after the intervention (*T*_3_). The study flowchart is presented in Figure 1.

### 2.2. Participants

Participants aged 40–65 years with a confirmed diagnosis of symptomatic degenerative RC tear by an orthopedic surgeon through clinical evaluation and magnetic resonance imaging (according to the criteria of the American Academy of Orthopaedic Surgeons Guideline) [26], treated with an arthroscopic RC repair, were included. They were able to read and understand Spanish. Participants were excluded if they had one of the following: massive irreparable RC tears, concomitant fracture, labral or nerve injury, suspicion of developing/diagnosis a frozen shoulder, revision surgery after RC repair, previous corticosteroid injection (< 1 year), recent surgery (< 1 year) in the contralateral shoulder, a history of deep venous thrombosis/pulmonary embolism, peripheral vascular disease, thrombophilia or clotting disorders, severe or uncontrolled hypertension, or any comorbid condition that prevented participants from complete the intervention [19]. Participants were asked not to start any other intervention, including exercise programs while participating in the study.

### 2.3. Recruitment

This study was carried out at the RedSalud Clinic, located in the Araucanía region, Chile. Participants were recruited, and data were collected between May and July 2025 from the Physical Therapy Unit. In the first session, a researcher not involved in the assessments determined which patients met the eligibility criteria and informed them about the objectives and all the details of the study. This process was supported by a recruitment script written by the research team. Patients who met the selection criteria were invited to participate and signed an informed consent prior to study participation.

### 2.4. Randomization, Allocation Concealment, and Blinding

Participants were randomized into two sequences of intervention (AB and BA) using a 1:1 ratio, where intervention A was isometric exercise alone and intervention B was isometric exercise with BFR. The randomization sequence was generated by a research assistant not involved in participant assessment using a centralized computer‐generated random number (https://www.random.org). This assistant then used sequentially numbered, opaque, and sealed envelopes for allocation concealment. During the first session, the treating physical therapist opened the envelope, viewed the assigned sequence, and applied intervention A or B, as appropriate. The statistician and the outcome assessor were blinded to the sequence allocation, data collection, and analysis of the participants. Unblinding was allowed only at the end of the study. Participants were not blinded to the allocated treatment due to the nature of the study design and experimental intervention. The physical therapists who performed the interventions were not blinded to the allocated treatment, as they needed to know which intervention was applied in each sequence.

### 2.5. Interventions

After arthroscopic surgery, participants were immobilized in a fixed sling for 2 weeks. During the third postoperative week, participants completed two sessions in the Physical Therapy Unit of the RedSalud Clinic. All participants performed the same evidence‐based isometric exercise protocol [4, 27]. The three isometric exercises of the shoulder were (i) external rotation, (ii) abduction, and (iii) flexion. Both intervention A (control) and intervention B (experimental) were performed with the same dose, and the only difference was the addition of BFR.

All participants underwent the interventions during the first and second sessions, which lasted 60 min each. At the first session, participants were randomly assigned to one of two intervention sequences (AB and BA). Participants assigned to the AB sequence performed isometric exercises alone (intervention A, control) in the first session and isometric exercises with BFR (intervention B, experimental) in the second session, while participants assigned to the BA sequence performed isometric exercises with BFR (intervention B, experimental) in the first session and isometric exercises alone (intervention A, control) in the second session.

#### 2.5.1. Isometric Exercises Alone (Intervention A, Control)

Three isometric exercises were performed following the recommendations of Kjær et al. [4]. Each of the three shoulder isometric exercises was performed for 10 repetitions, with a 15‐s isometric contraction, followed by a 15‐s rest period. The low‐load intensity during isometric exercises was calculated in the unaffected upper limb before each of the three exercises and in the same position using the Tindeq Progressor load cell (Tindeq, Norway). During each exercise, the participant received real‐time visual feedback to ensure they achieved an intensity close to 20%–25% of the maximal voluntary isometric contraction (MVIC) [28]. The rest period after each exercise was 2 min, and the pain level was maintained below 5 on the verbal Numeric Pain Rating Scale (NPRS) [4]. This pain intensity threshold was defined to ensure safety and avoid pain flares. Any pain exceeding this threshold was addressed by immediately adjusting the range of motion or the MVIC.

#### 2.5.2. Isometric Exercises With BFR (Intervention B, Experimental)

**FIGURE 2:**
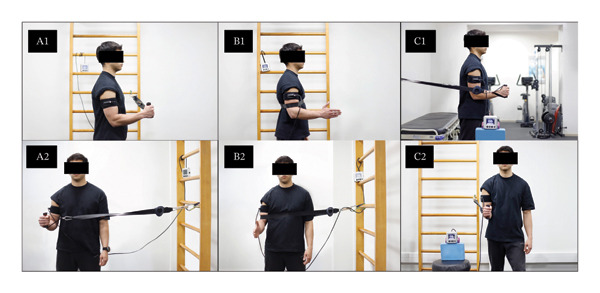
Representative picture of postoperative isometric exercises with blood flow restriction: isometric shoulder external rotation (A) in lateral (A1) and frontal (A2) views, isometric shoulder abduction (B) in lateral (B1) and frontal (B2) views, and isometric shoulder flexion (C) in lateral (C1) and frontal (C2) views.

The three shoulder isometric exercises were performed with the addition of BFR following the recommendations of Patterson et al. [19] and Kjær et al. [4]. The BFR was achieved with a self‐regulated portable SmartCuffs® 3.0 Pro system (Smart Tools Plus, Strongsville, Ohio, USA), with a 17‐inch‐long and 5‐inch‐wide cuff positioned at the level of deltoid tuberosity on the proximal arm. Each session began with a maximal occlusion test to customize the restriction pressure during the intervention. Each exercise used a self‐regulated arterial occlusion pressure (AOP) at 60% of the maximal occlusion pressure, which was calculated using the automatic SmartCuffs algorithm. Each isometric exercise with BFR was performed for 10 repetitions, with a 15‐s isometric contraction, low‐load intensity close to 20%–25% of MVIC [28], followed by a 15‐s rest period. After each exercise, the cuff was deflated (reperfusion) and the participant rested for 2 minutes. During session, participants were asked to perform the three isometric BFR exercises with a pain level below 5 on the NPRS [4]. The physical therapists responsible for the application of the exercise intervention were trained on how to implement the postoperative shoulder isometric BFR exercise protocol, including the pain intensity allowed and the exercise dose. All details of the isometric exercises with BFR are shown in Figure 2.

### 2.6. Data Collection

In the first session, the initial assessment (*T*_1_) was conducted, in which participants completed the screening criteria, recorded biodemographic data, and performed clinical measurements. All questionnaires were submitted in printed form and answered individually in writing. Immediately (*T*_2_) and 10 min (*T*_3_) after the intervention (A or B), the acute effect was assessed by evaluating clinical variables. In the second session, after a 72‐h washout period, clinical variables were measured to determine whether there was a carryover effect after the washout period and before exercise (*T*_1_); immediately (*T*_2_) and 10 min (*T*_3_) after the intervention (A or B), the acute effect was assessed by evaluating clinical variables. In addition to the washout period, the first and second sessions were conducted at the same time of day to ensure minimal impact from prior exposure, allow recovery from exercise, and limit daytime influences. All participants were instructed to continue using the analgesics prescribed by their treating surgeon, as well as maintain similar levels of physical activity between visits to reduce the impact of these factors on the study.

To standardize the protocols, the outcome assessors and the treating physical therapist were trained in all assessment and treatment procedures prior to the start of the study. Measurement information was recorded in a specially designed clinical record. All assessments were filed in a folder and stored in a locked cabinet, accessible only to the principal researcher.

### 2.7. Outcome Measures

#### 2.7.1. Primary Outcomes

PPT: Pain sensitivity was assessed bilaterally by PPT (measured in kg/cm^2^) using a Wagner FPX25 pressure algometer on the deltoid muscle (10 cm lateral to the acromion) and the upper trapezius muscle (halfway between the acromion and C7). Three measurements separated by 30 s were taken in each structure, and the average value was considered for analysis. Pressure algometry was shown to be a reliable tool for measuring PPT in patients with shoulder pain. A minimum detectable change (MDC) of > 1.1 kg/cm^2^/s was reported [29]. The intrarater reliability (intraclass correlation coefficient [ICC]) of the outcome assessors for PPT levels was calculated for both sides, which was determined from 3 trials collected in 20 healthy adults before the recruitment in the study. The ICC was 0.973 for deltoid and 0.957 for upper trapezius on the affected side and was 0.985 for deltoid and 0.961 for upper trapezius on the unaffected side, suggesting an excellent repeatability of PPT testing.

CPM: This paradigm was used to assess the integrity of endogenous analgesic mechanisms that had been linked to the hypoalgesic response to exercise [30]. As a conditioning stimulus, the hand contralateral to the operated shoulder was immersed for 1 min in cold water (10°C) [31]. PPT was assessed in the bilateral deltoid and trapezius muscles before, during, and after the conditioning stimulus. The “during” assessment was performed when the participant reported a pain intensity of 6/10 on the NPRS in the immersed limb. For this, the participant was asked about the intensity of the pain every 30 s the first time and every 15 s the following times, up to a maximum immersion time of 2 min. Using a cold thermal stimulus, the conditioned pain assessment showed excellent intrasession reliability, with an ICC of 0.85 [32]. The percentage of change (CPM effect) was calculated using the following formula [33]: [(PPT post − PPT pre)/PPT pre] × 100.

#### 2.7.2. Secondary Outcome

Pain intensity: The visual analog scale (VAS), a valid and reliable tool, was used to assess pain intensity at rest and during movement. The VAS consisted of a 10‐cm horizontal line, where the range was defined from 0 (“no pain”) to 10 (“worst pain imaginable”). This scale has a reported MCID of 2.2 cm for shoulder pain [34].

#### 2.7.3. Covariates

Kinesiophobia: It was measured with the short version of the Tampa Scale of Kinesiophobia (TSK‐11). This self‐reported 44‐point instrument was shown to be valid and reliable [35].

Shoulder pain–related disability: The Shoulder Pain Disability Index (SPADI) was used. This questionnaire was developed to assess the pain and disability associated with shoulder dysfunction [36]. SPADI consists of 13 items on a patient’s ability to perform basic activities of daily living. Each item is rated using a numerical rating scale ranging from 0 (no pain/no difficulty) to 10 (worst pain imaginable/most difficulty). The SPADI provides a pain domain (5 items; scale score range 0–50 points, expressed as a percentage) and a disability domain (8 items; scale score range 0–80 points, expressed as a percentage). The scores for the two domains were averaged to give a summary score ranging from 0% to 100%, with a higher score indicating greater shoulder pain–related disability [36]. The version adapted and validated to Spanish [37] was used.

Quality of life: The Western Ontario Rotator Cuff (WORC) index was used to assess the impact of a RC tear on an individual’s quality of life [38]. It consists of 21 questions, with answers reported on a 100‐mm VAS; questions address areas such as perceived responses to physical symptoms, sport and recreation, work, lifestyle, and emotions. The final score is reported as a percentage, and higher scores are associated with fewer symptoms [38]. The adapted and validated Spanish version was used [39].

### 2.8. Adverse Events or Side Effects

Participants were informed of the potential side effects resulting from the BFR intervention, including instructions on how to proceed if they occur. All participants were informed that they may experience mild side effects, such as numbness, a cold sensation, or minor bruising in the application area that often resolve in less than 24 h [25]. Furthermore, the participants were informed that this intervention could cause serious adverse events, although the probability is very low (deep vein thrombosis: 0.06%, pulmonary embolism: 0.008%, and rhabdomyolysis: 0.008%) [40]. In each supervised session, the participant was asked to report any side effects or adverse events.

### 2.9. Training and Fidelity

The physical therapists responsible for implementing the interventions were trained by a physical therapist (F‐PF) with more than 15 years of clinical experience in musculoskeletal rehabilitation and certified in BFR training. Furthermore, the outcome assessors responsible for evaluating all outcome variables were trained on how to implement the participants’ eligibility criteria, appropriate assessment of outcome measures, and correctly record the data.

### 2.10. Data Analysis

#### 2.10.1. Sample Size

A sample size calculation was performed using GPower 3.1 software, based on a repeated measures ANOVA test for difference in means, two groups, three measurements, alpha risk of 0.05, and statistical power of 0.80. The effect size for PPT was based on a previous BFR training study [25] (Cohen’s *d* = 0.69), which was converted to an effect size *f* of 0.345 for the GPower input. Assuming a correlation among repeated measures of 0.5 (default), the required total sample size was 18 participants, which yields an actual power of 0.849. Considering a 20% dropout rate, the sample size was 23 participants.

#### 2.10.2. Data Analysis

An intention‐to‐treat analysis was performed for all randomly assigned participants. All randomized participants completed all follow‐up assessments; therefore, no imputation for missing data was necessary. Analyses were performed using IBM SPSS Statistics version 24.0. Data normality was assessed using the Shapiro–Wilk test and homogeneity of variance was assessed using the Levene test. Furthermore, the assumption of sphericity was assessed using Mauchly’s test; if the assumption was violated (*p* < 0.05), the Greenhouse–Geisser correction was applied to the degrees of freedom. Descriptive statistics were used to summarize the data for continuous variables and are presented as mean ± SD, 95% confidence interval (CI). For differences between groups, a two‐way repeated measures analysis of variance (ANOVA) was used to determine the effect of the factor “treatment group” within participants in each of the two categories (isometric exercise with BFR and isometric exercise alone) and the factor “time” within participants in each of the three categories (pre‐, post‐0, and post‐10 min). The inclusion of TSK‐11, SPADI, and WORC scores as covariates in the models was analyzed. A post hoc analysis with Bonferroni correction was performed for significant findings of the ANOVA for multiple comparisons between variables. The results of the PPT (kg/cm^2^) and CPM (%) variables were interpreted using the change or increment (Δ) between the measurements and the effect size (partial eta‐squared, $n_{p}^{2}$). To determine the magnitude of the effects, $n_{p}^{2}$ was calculated for the ANOVA interactions and interpreted using Cohen’s cut‐off values: $n_{p}^{2}$ ≈ 0.01 indicates a small effect, $n_{p}^{2}$ ≈ 0.06 a medium effect, and $n_{p}^{2}$ ≥ 0.14 a large effect [41]. A Pearson correlation (*r*) was performed to analyze the relationship between the variables Δ PPT and Δ CPM with the results of the TSK‐11, SPADI, and WORC questionnaires, after confirming the variables met the assumptions for normality using the Shapiro–Wilk test. The values of the correlation coefficient were considered as follows: close to 0 as no correlation, 0.1–0.3 as weak, 0.4–0.6 as moderate, 0.7–0.9 as strong, and 1 as perfect correlation [42]. Carryover effect between sessions for PPT and CPM differences was evaluated according to a mixed‐model ANOVA, including “Sequence” as a between‐subjects factor and “Time” as a within‐subjects factor. The level of significance was established at *p* ≤ 0.05.

## 3. Results

A total of 23 adult participants (11 women), with a mean age of 58.1 years and repaired RC participated in the study. Baseline demographic and clinical data are shown in Table 1. No adverse effects were reported during or after the experimental sessions.

**TABLE 1:** Baseline demographic and clinical data (*n* = 23).

	Mean	SD	CI_95%_	
			Lower limit	Upper limit
Age, y	58.1	6.6	55.9	59.6
Weight, kg	83.0	18.7	77.4	88.5
Height, cm	162.8	10.0	160.0	165.6
BMI	31.3	6.7	29.3	33.3
Pain duration, months	7.7	9.5	4.8	10.5
TSK‐11	23.3	8.5	20.7	25.8
SPADI pain subscale, %	41.3	9.5	38.4	44.1
SPADI function subscale, %	60.1	16.0	55.4	64.9
SPADI total score, %	101.4	24.2	94.2	108.6
WORC physical symptoms subscale, %	32.1	11.8	28.6	35.6
WORC sports and recreation subscale, %	29.7	6.1	27.9	31.5
WORC work subscale, %	30.6	6.2	28.8	32.5
WORC lifestyle subscale, %	30.0	6.7	27.0	30.9
WORC emotions subscale, %	20.1	5.5	18.5	21.7
WORC total score, %	32.6	14.6	28.3	36.9

*Note:* CI_95%_, 95% confidence interval; TSK‐11, 11‐item Tampa Scale for Kinesiophobia; WORC, Western Ontario Rotator Cuff index.

Abbreviations: BMI, body mass index; SPADI, shoulder pain and disability index.

The descriptive statistics (mean ± SD) for the primary outcome variables (PPT and CPM), at all time points (pre‐, post‐0, and post‐10 min) for both intervention groups are presented in Tables 2 and 3, respectively. As a preliminary analysis, no significant differences were detected at baseline values between both intervention groups for the PPT in the affected (deltoid: *p* = 0.059; upper trapezius: *p* = 0.919) and unaffected limb (deltoid: *p* = 0.293; upper trapezius: *p* = 0.328), and CPM in the affected (deltoid: *p* = 0.173; upper trapezius: *p* = 0.200) and unaffected limb (deltoid: *p* = 0.178; upper trapezius: *p* = 0.502). No significant carryover effect was detected for these variables, suggesting independence between study periods (*p* > 0.05) (Supporting 1).

**TABLE 2:** PPT results of the affected and unaffected limbs of the participants.

Limb	Measure	Group	Mean ± SD, kg/cm^2^			Mean between‐group difference (CI_95%_). (a) post‐immediate; (b) post‐10 min.
			Pre	Post	Post‐10 min	
Affected	Deltoid PPT, kg/cm^2^	Control	2.8 ± 1.0	2.3 ± 1.1	3.0 ± 1.2	(a): **−0.6** (−1.3 to 0.1); (b): 0.1 (−0.6–0.8)
		BFR	2.5 ± 1.2	2.9 ± 1.3	2.9 ± 1.2	
	Upper trapezius PPT, kg/cm^2^	Control	2.8 ± 1.4	2.8 ± 1.2	3.1 ± 1.4	(a): **−0.4** (−1.2 to 0.4); (b): −0.1 (−0.9 to 0.7)
		BFR	2.8 ± 1.1	3.2 ± 1.4	3.2 ± 1.2	

Unaffected	Deltoid PPT, kg/cm^2^	Control	3.0 ± 1.5	3.0 ± 1.2	2.8 ± 1.4	(a): −0.1 (−0.8 to 0.6); (b): 0.3 (−1.1 to 0.5)
		BFR	2.7 ± 1.2	3.1 ± 1.1	3.1 ± 1.4	
	Upper trapezius PPT, kg/cm^2^	Control	3.1 ± 1.3	3.5 ± 1.3	3.1 ± 1.3	(a): 0.3 (−0.4–1.0); (b): 0.0 (−0.7 to 0.7)
		BFR	2.9 ± 1.1	3.2 ± 1.1	3.1 ± 1.2	

*Note:* CI_95%_, 95% confidence interval; control group, isometric exercises alone; experimental group, isometric exercises with BFR. Bold, statistically significant difference (*p* < 0.05).

Abbreviation: PPT, pain pressure threshold.

**TABLE 3:** CPM results of the affected and unaffected limbs, and VAS at rest and movement of the participants.

Limb	Measure	Group	Mean ± SD, kg/cm^2^			Mean between‐group difference (CI_95%_).
			Pre	Post	Post‐10 min	(a) post‐immediate; (b) post‐10 min.
Affected	Deltoid CPM, %	Control	6.2 ± 26.2	36.3 ± 44.3	−3.5 ± 18.2	(a): **29.6** (7.7–51.5); (b): 2.4 (−7.8–12.6)
		BFR	17.5 ± 32.2	6.7 ± 30.2	−5.9 ± 17.4	
	Upper trapezius CPM, %	Control	65.8 ± 189.4	13.3 ± 20.4	1.6 ± 21.0	(a): 10.6 (0.2–21.0); (b): 1.8 (−9.0–12.6)
		BFR	13.6 ± 10.7	2.7 ± 15.7	−0.2 ± 16.1	

Unaffected	Deltoid CPM, %	Control	7.6 ± 23.0	0.7 ± 15.6	18.2 ± 30.8	(a): 1.7 (−6.6–10.0); (b): **14.3** (−0.6–29.2)
		BFR	15.9 ± 21.8	−1.0 ± 13.4	3.9 ± 19.6	
	Upper trapezius CPM, %	Control	14.5 ± 25.0	−4.9 ± 15.4	9.3 ± 21.9	(a): −6.8 (−16.2 to 2.6); (b): 4.1 (−9.0–17.2)
		BFR	11.0 ± 14.4	1.9 ± 17.2	5.2 ± 23.1	

NA	VAS at rest, cm	Control	2.6 ± 2.2	2.9 ± 1.9	2.4 ± 2.3	(a): −0.3 (−1.6 to 1.0); (b): −0.4 (−1.7 to 0.9)
		BFR	2.9 ± 2.3	3.2 ± 2.4	2.8 ± 2.2	

NA	VAS at movement, cm	Control	5.7 ± 2.3	5.2 ± 3.0	4.5 ± 2.4	(a): 0.4 (−1.2–2.0); (b): 0.0 (−1.31 to 1.31)
		BFR	5.5 ± 2.2	4.8 ± 2.5	4.5 ± 2.1	

*Note:* CI_95%_, 95% confidence interval; control group, isometric exercises alone; experimental group, isometric exercises with BFR. Bold, statistically significant difference (*p* < 0.05).

Abbreviations: CPM, conditioned pain modulation; NA, not applicable; VAS, visual analog scale.

**FIGURE 3:**
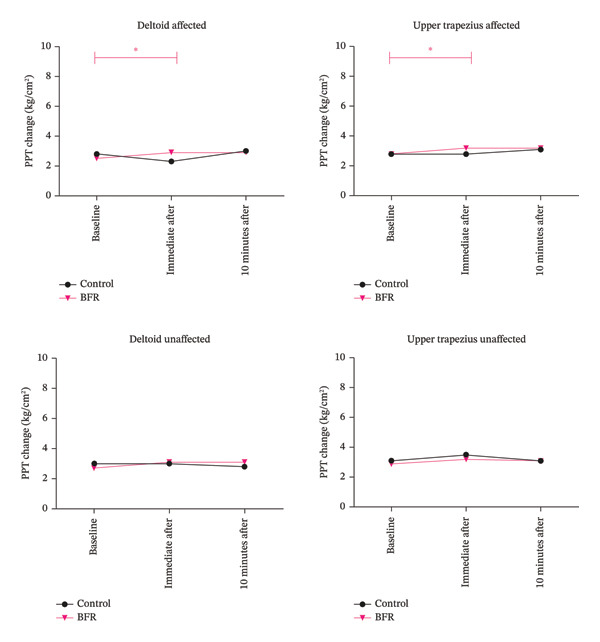
Changes in PPT over time. Mean PPT change (%) at baseline, immediately after, and 10 min after intervention for the control (isometric exercise alone) and BFR (isometric exercise with BFR) groups. Panels display data from the deltoid and upper trapezius on both the affected and nonaffected limbs. Asterisks denote significant time‐by‐group interactions (^∗^ *p* < 0.05, ^∗∗^ *p* < 0.01). BFR, blood flow restriction.

The two‐way repeated measures ANOVA for PPT revealed a significant time‐by‐group interaction between two groups in the deltoid (affected_pre-post_: *F* = 4.414, *p* = 0.005, $n_{p}^{2}$ = 0.301; unaffected_pre-post_: *F* = 5.388, *p* = 0.025, $n_{p}^{2}$ = 0.116) and upper trapezius (affected_pre-post_: *F* = 4.301, *p* = 0.044, $n_{p}^{2}$ = 0.095). Post hoc between‐group comparisons (detailed in Table 2 and Figure 3) demonstrated that the BFR isometric exercises achieved higher PPT values than the isometric exercises alone immediately after exercise in the affected upper trapezius (MD: −0.4 kg/cm^2^; CI_95%_ −1.2 to 0.4). Similarly, in the unaffected limb, the BFR isometric exercises showed superior PPT levels in the deltoid compared to the isometric exercises alone post‐10 min intervention (MD: 0.3 kg/cm^2^; CI_95%_ −1.1 to 0.5). Critically, none of these between‐group comparisons reached statistical significance as all CIs crossed zero, and the absolute changes did not reach the MCID threshold in either group. Additionally, while the SPADI score showed a significant main effect on PPT (*p* = 0.034), it did not act as a moderator for the treatment effect, suggesting that baseline disability influenced overall pain sensitivity but not the specific hypoalgesic response triggered by BFR exercise.

**FIGURE 4:**
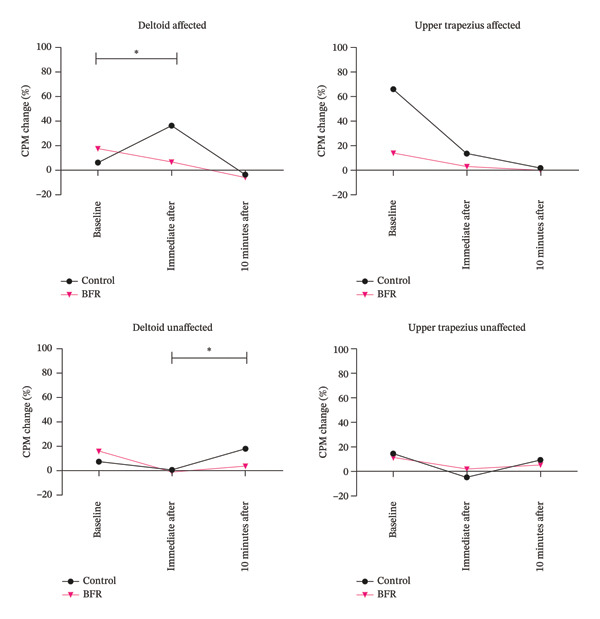
Changes in CPM over time. Mean CPM change (%) at baseline, immediately after, and 10 min after intervention for the control (isometric exercise alone) and BFR (isometric exercise with BFR) groups. Panels display data from the deltoid and upper trapezius on both the affected and nonaffected limbs. Asterisks denote significant time‐by‐group interactions (^∗^ *p* < 0.05, ^∗∗^ *p* < 0.01). BFR, blood flow restriction.

For the CPM analysis, the two‐way repeated measures ANOVA revealed significant group‐by‐time interactions for the deltoid on the affected limb (pre‐post: *F* = 9.149, *p* = 0.004, $n_{p}^{2}$ = 0.182; post‐post_10_: *F* = 5.041, *p* = 0.030, $n_{p}^{2}$ = 0.109) and the unaffected limb (pre‐post_10_: *F* = 6.773, *p* = 0.013, $n_{p}^{2}$ = 0.142). Post hoc between‐group comparisons (detailed in Table 3 and Figure 4) showed that at 10‐min follow‐up, the isometric exercises alone exhibited significantly higher CPM efficiency in the unaffected deltoid compared to the BFR isometric exercises (MD: 12.0%; CI_95%_ −0.4–24.4). Similarly, immediately after the intervention, the isometric exercises alone showed higher CPM values in the affected deltoid compared with the BFR isometric exercises (MD: 30.1%; CI_95%_ 10.8–49.4).

There were no differences in VAS scores at rest (pre‐post: *F* = 0.242, *p* = 0.625, $n_{p}^{2}$ = 0.006; post‐post10: *F* = 0.342, *p* = 0.562, $n_{p}^{2}$ = 0.008; pre‐post10: *F* = 0.435, *p* = 0.513, $n_{p}^{2}$ = 0.010) and during movement (pre‐post: *F* = 0.186, *p* = 0.668, $n_{p}^{2}$ = 0.005; post‐post10: *F* = 0.510, *p* = 0.479, $n_{p}^{2}$ = 0.012; pre‐post10: *F* = 0.000, *p* = 0.995, $n_{p}^{2}$ = 0.000) between isometric exercises alone and isometric exercises with BFR.

The covariate analysis found that the baseline SPADI score had a significant effect on the PPT (*p* = 0.034), specifically in the affected upper trapezius muscle. This means that the baseline level of pain and disability influenced how much pain sensitivity changed after exercise. Furthermore, it was found that WORC score had a significant effect on the VAS at rest and movement (*p* < 0.05), indicating that baseline quality of life reported by patients influenced how much their pain intensity changed after exercise.

Regarding the relationship between psychological and clinical factors and pain modulation, no associations were found between kinesiophobia (TSK‐11) and EIH measures (*p* > 0.05). Furthermore, most clinical baseline variables showed no significant relationship with changes in PPT or CPM. As shown in Table 4, only a few weak negative correlations were observed between PPT changes in the affected deltoid and baseline WORC scores (sport: *r* = −0.322, *p* = 0.029; work: *r* = −0.331, *p* = 0.025; lifestyle: *r* = −0.302, *p* = 0.041), as well as between PPT changes in the unaffected upper trapezius and baseline SPADI pain (*r* = −0.309, *p* = 0.037). Given the low magnitude of these coefficients, these findings suggest a limited association between baseline clinical status and the acute EIH response.

**TABLE 4:** Correlation analysis between covariates using Pearson coefficient (*r*).

Limb	Variables	TSK‐11	SPADI pain	SPADI total	WORC sport	WORC work	WORC lifestyle	WORC total
Affected	Δ PPT deltoid	−0.040	−0.254	−0.107	**−0.322**	**−0.331**	**−0.302**	−0.239
	Δ PPT upper trapezius	0.082	−0.196	−0.118	−0.009	−0.012	0.059	0.055
	Δ CMP deltoid	0.146	0.133	0.073	0.110	0.177	0.159	0.141
	Δ CPM upper trapezius	−0.101	−0.016	−0.026	−0.138	−0.104	−0.211	−0.126

Unaffected	Δ PPT deltoid	0.124	0.001	0.057	0.095	−0.006	0.099	0.055
	Δ PPT upper trapezius	0.039	**−0.309**	−0.230	−0.113	−0.075	−0.114	−0.066
	Δ CPM deltoid	0.047	−0.204	−0.213	−0.231	−0.088	−0.163	−0.165
	Δ CPM upper trapezius	−0.129	0.087	0.060	−0.071	0.014	−0.149	−0.079

*Note:* Δ, change between pre and post measurements; TSK‐11, 11‐item Tampa Scale for Kinesiophobia; WORC, Western Ontario Rotator Cuff index. Bold, statistically significant (*p* < 0.05).

Abbreviations: CPM, conditioned pain modulation; PPT, pressure pain thresholds; SPADI, shoulder pain and disability index.

## 4. Discussion

The main finding of this study was that although a single session of isometric exercise with BFR resulted in modest acute improvements in pain sensitivity (PPT) within the experimental condition, these changes were not significantly superior to those observed with isometric exercise alone. Furthermore, the observed effects remained below clinically meaningful thresholds for patients following arthroscopic RC repair. Consequently, no consistent between‐group differences were demonstrated at any time point for all variables, suggesting that the addition of BFR does not provide an immediate superior hypoalgesic advantage over conventional isometric exercise in this population. In addition, the descending pain modulation (CPM) response was inconsistent across both conditions. Notably, the control group demonstrated a more robust increase in CPM efficiency in the affected limb immediately after exercise compared to the BFR group. However, the high variability and CIs crossing zero for both interventions highlight the lack of a reliable mechanistic pathway. These findings suggest that neither isometric exercise alone nor with BFR consistently triggers the descending inhibitory system in patients with RC repair.

### 4.1. Changes in Pain Sensitivity

Findings from the present study demonstrate that low‐intensity isometric exercises with BFR do not produce an acute EIH in patients following RC repair. Although the BFR group showed internal improvements in PPT, it is important to note that these changes were similar in magnitude to those observed in the control group, suggesting that the addition of BFR might not significantly augment the acute hypoalgesic effect of isometric exercise alone in this population. This is comparable with findings in asymptomatic people [13] and aligns with the variable responses seen in musculoskeletal pain populations [43], although some conditions like fibromyalgia may cause hyperalgesia [44]. Three studies investigated the effects of isometric exercises on PPT in shoulder pain patients [28, 45, 46]. Kuppens et al. [28] found a significantly increased PPT (augmented EIH) at the ipsilateral and contralateral shoulder in musicians after isometric abduction (20%–25% MVIC) in the painful shoulder [28]. Similarly, Persson et al. [45] found an increase in the shoulder PPT after isometric shoulder abduction exercise in chronic shoulder pain patients [45]. Lannersten and Kosek [46] observed that patients with shoulder myalgia experienced increased shoulder PPT (augmented EIH) when performing knee extensions with their nonpainful leg. However, when the exercise was executed by the painful shoulder (isometric shoulder external rotation at 20%–25% of MVIC), no hypoalgesic response was observed [46]. This contrasts with the acute hypoalgesic effect observed after isometric exercises with BFR in the painful shoulder in our study. The increase in PPT (EIH response) in our study was not clinically significant, which contrasts with studies on other painful musculoskeletal populations, particularly those with knee pain [16–18, 47]. For instance, Korakakis et al. [16] found that a single bout of low‐load BFR exercise induced a clinically significant hypoalgesic effect in patients with anterior knee pain, an effect that was sustained for at least 45 min post‐intervention. A subsequent RCT by the same authors [17] indicated that low‐load BFR exercise produced significant immediate pain reduction (with large effect sizes). Similarly, Constantinou et al. demonstrated that BFR exercise could produce short‐term pain reduction in individuals with patellofemoral pain [22]. Critically, 20% of the non‐BFR group in Korakakis et al.’s study [17] reported a worsening of symptoms, compared to 0% in the BFR group, suggesting BFR provides a more reliable analgesic window. Furthermore, in patients with end‐stage knee osteoarthritis, Ogrezeanu et al. [18] found that 80% AOP BFR improved objective PPT (hypoalgesia) locally, contralaterally, and systemically, even while increasing subjective VAS scores, which was likely due to cuff discomfort. While these diverse musculoskeletal cohorts provide a useful framework, our findings indicate that BFR did not demonstrate a robust or consistent statistical superiority over the control group across time points in the RC repair postoperative setting. While these diverse musculoskeletal cohorts provide a useful framework for understanding BFR‐induced pain modulation, it is important to note that these findings may not be directly transferable to the specific biological and irritability profiles of patients in the early stages of RC repair.

There are several possible explanations for the findings of our study. First, it is well acknowledged that the intensity of exercise is an important determinant of the magnitude of EIH, with higher‐intensity exercise resulting in greater EIH [48]. The addition of BFR to low‐intensity isometric exercises augmented the effort perception of the patients during exercises, which may facilitate a greater EIH and may be a more tolerable alternative in RC postoperative rehabilitation context. Second, it has been observed that discomfort and pain are exacerbated during BFR [49] and mediates the relationship of exercise intervention on change in PPT [25]. This may suggest a higher discomfort generated during low‐load isometric exercises with BFR led to greater EIH response [50]. Third, it has been shown that there is a greater increase in PPT after a long‐duration isometric contraction [51] and a greater volume of exercise [52]. In this sense, longer isometric contraction schemes (19 min in total) used in our study may have contributed to greater EIH among the patients. Fourth, the use of an MDC threshold (> 1.1 kg/cm^2^/s) validated for acute neck pain represents a potential limitation when assessing a postoperative RC cohort. The direct extrapolation of this criterion might not be appropriate, given the distinct pathophysiological and functional characteristics between the two populations. This methodological approach could explain our failure to identify clinically meaningful changes, suggesting the need for population‐specific thresholds in future research. Regardless of the statistical interactions observed, the lack of clinical significance further supports the interpretation that there is no clear therapeutic advantage of one protocol over the other regarding acute pain modulation in this clinical setting.

In patients with chronic pain, studies have suggested impaired EIH after isometric exercise compared with asymptomatic controls [46]. In fact, hyperalgesia after exercise is observed in individuals with more widespread chronic pain conditions [44]. Considering that 78% of our sample were patients with chronic pain, it is surprising that the greater acute EIH after isometric exercises with BFR. Due to the psychological factors influencing EIH response [53], it is likely that low kinesiophobia score at baseline in our patients acted as a protective factor. Furthermore, in chronic pain populations the magnitude and direction of the EIH is highly variable after isometric contractions and appears to depend on the chronic pain condition being studied as well as the intensity of the exercise [13]. Regarding the correlation between baseline status and pain modulation, our findings revealed only weak negative correlations between PPT changes and baseline WORC or SPADI scores. This indicates that although baseline disability influenced overall pain sensitivity, these clinical factors explain only a small fraction of the EIH variance. Consequently, the acute hypoalgesic response triggered by BFR exercise appears to be largely independent of initial clinical severity, suggesting that the physiological stimulus of the intervention may override baseline clinical traits in this early postoperative phase.

### 4.2. Changes in Pain Modulation

Given the changes in PPT observed, it is also important to examine the results obtained through the CPM paradigm. The descending pain modulation response was inconsistent across both study conditions. In fact, the control group demonstrated a more robust increase in CPM efficiency in the affected limb immediately after exercise compared to the BFR group. The acute shifts in CPM after isometric exercise with BFR found in our study are only partially in line with previous studies. Preliminary evidence suggests that shoulder pain may be associated with impaired CPM [54], although other evidence suggests that CPM is not necessarily impaired in individuals with frozen shoulder [55] or shoulder myalgia [46].

In our study, the deltoid CPM in the unaffected limb showed a significant statistical increase 10 min after BFR exercise; however, the CI crossed zero (CI_95%_ −0.4–24.4), and the effect was small. Similarly, while isometric exercise alone increased deltoid CPM immediately after in the affected limb (MD: 30.1 [CI_95%_ 10.8 to 49.4]), this does not demonstrate a consistent mechanistic pathway. These findings could be influenced not only by the descending pain modulation system [56], but also by other factors such as the use of opioids [57], level of kinesiophobia [58], expectations [59], and aging [60]. Specifically, a higher level of kinesiophobia is generally associated with a lower hypoalgesic effect [53]; thus, in the context of our crossover design, where psychological traits remain constant across both interventions, the overall low levels of kinesiophobia in our patients could function as a baseline protective factor, facilitating the observed CPM results in both conditions rather than favoring one over the other. In addition, positive expectations are associated with higher hypoalgesic responses [61]. RC repair may be associated with high positive expectations [62], which might have influenced the CPM results in our study. However, this remains speculative, as these contextual factors were not directly controlled. Although we observed an acute increase in CPM despite the advanced age of our patients [60], a definitive explanation regarding the underlying mechanism cannot be established due to the lack of biochemical data and the presence of potential confounders, such as the painful nature of the conditioning stimulus.

### 4.3. Potential Mechanisms of EIH

There are likely multiple mechanisms that are responsible for EIH [63]. In the context of our study, a hypothetical mechanism by which low‐intensity isometric exercise with BFR might trigger hypoalgesia is through potentially facilitation of the opioid and endocannabinoid mechanisms. During BFR exercise, the occlusion pressure restricts venous blood flow to the upper limb [64], creating a local ischemic‐hypoxia state and increasing muscle metabolic stress [65]. Elevated concentrations of metabolites may stimulate muscle chemoreceptors [66], potentially activating group III and IV nociceptive fibers [67], which could alter afferent feedback to the central nervous system. Furthermore, it has been suggested in the literature that metabolite‐induced stimulation of nociceptive fibers and increased sympathetic nervous activity [68] result in an increased perception of pain/discomfort, which is thought to potentially trigger opioid and endocannabinoid pathways [56]. The shifts in CPM found in our study do not provide evidence of these pathways, especially as the discomfort during BFR exercises may act as a noxious conditioning stimulus itself. Furthermore, as this study did not include biochemical data or plasma markers to confirm the release of endogenous substances, these mechanistic links remain entirely speculative. As CPM uniquely predicted only 8.8% of the variance in EIH [69], it is possible that other mechanisms, such as threshold motor unit recruitment or serotonergic and immune systems, act simultaneously. Additionally, our results indicated that baseline kinesiophobia and quality of life did not moderate the EIH response. This suggests that the potent physiological nature of BFR exercise could potentially mitigate the expected inhibitory effects of psychological traits in this early postoperative stage. It is possible that the physiological stimulus provided by BFR exercise might be sufficiently robust to partially counteract the potential inhibitory influence of psychological factors on endogenous pain modulation, aligning with previous observations where psychological traits did not necessarily correlate with acute EIH changes [15, 70]. Therefore, the observed hypoalgesic response must be interpreted strictly as a net behavioral effect that was not superior to the control condition, rather than a confirmed physiological pathway.

### 4.4. Implications in Clinical Practice

From a clinical perspective, our findings provide preliminary insights into BFR dosing and timing during early RC postoperative rehabilitation. While we observed an acute statistical signal within the BFR group, it is essential to note that this intervention did not demonstrate clinically meaningful hypoalgesia, nor was it superior to isometric exercise alone. This acute statistical signal of EIH observed after isometric exercises with BFR theoretically provides a potential temporary therapeutic window that might facilitate earlier active exercise. However, this hypothesis remains to be tested in longitudinal studies measuring physical variables and functional progression. By utilizing this acute reduction in pain sensitivity, clinicians could potentially assist in rehabilitation progression, although the long‐term impact on session‐to‐session tolerance was not evaluated in this trial. It is essential to note that our study found a statistical signal but did not demonstrate clinically meaningful hypoalgesia, as the changes in PPT did not exceed MDC. Therefore, integrating PPT and CPM assessments should be viewed as a way to monitor individual variability rather than as confirmation of an established analgesic mechanism. Clinicians should proceed with low‐load BFR for short‐term modulation while closely monitoring patient tolerance, regardless of inconsistent CPM shifts.

Future research should focus on longitudinal trials to determine if these acute responses translate into long‐term recovery. To reach clinically meaningful hypoalgesia (i.e., MDC), future studies should explore increasing exercise intensity (30%–35% MVIC) and occlusion pressure (60%–70% AOP), alongside multi‐session protocols and familiarization periods. Additionally, incorporating sham‐BFR groups and unaffected limb (cross‐education) applications may clarify the role of contextual and systemic factors. Finally, integrating biomarkers and psychophysical moderators will help determine whether BFR‐driven adaptations are peripheral, central, or systemic.

### 4.5. Strengths and Limitations

This study is the first to investigate EIH following isometric exercise with BFR in adults after arthroscopic RC repair. Nonetheless, several limitations should be acknowledged. First, the lack of standardization of the CPM assessment may limit comparability with other studies [71], albeit this was mitigated by a validated, widely used cold paradigm used in previous studies [72]. Second, although the washout period was based on a previous similar study [25] and the analysis confirming the absence of carryover effects, there is a possibility that a 72‐h washout period was too short for BFR training, as delayed onset muscle soreness can persist for up to 72 h [73]. In addition, a longer follow‐up (e.g., 30 or 45 min) was not possible due to limitations inherent in the demanding clinical context. Third, the treating physical therapists were not blinded to the allocated treatment, which may have introduced performance bias through differential enthusiasm or effort between interventions. Fourth, although fear‐avoidance was assessed (via TSK‐11), other critical psychological covariates such as catastrophizing [74], anxiety [75], and patient expectations [11] were not measured and which may influence the EIH response. The lack of blinding could potentially contribute to expectancy effects. However, the selective improvement in PPT and CPM, in contrast to the absence of change in VAS scores, provides a basis to consider a physiological component in the EIH response. It is plausible that a generalized placebo or expectancy response would have manifested as a more consistent trend across both clinical and experimental pain measures. In addition, although 78% of the participants met the criteria for chronic pain, a stratified analysis was not performed. Chronic pain status is known to significantly influence EIH and CPM responses [46]; however, the present study was not powered for subgroup analyses. Future research with larger cohorts is needed and we should consider incorporating sham‐BFR protocols and a broader battery of psychological scales to further refine our understanding of these effects. Fifth, the medications used were not strictly controlled, which could have influenced pain outcomes. Three orthopedic surgeons with similar pharmacological approaches referred to the patients, so the variability in medications used was possibly low. Additionally, the crossover nature of the study design, where each participant serves as their own control, partially mitigates this influence by ensuring that medication effects were relatively constant across both intervention arms. Sixth, other hypoalgesia mechanisms (i.e., threshold motor unit recruitment, serotonergic, and immune systems) were not evaluated in our study and may have contributed to the EIH response. In addition, our study lacked biochemical data to support any mechanistic interpretation involving opioid or endocannabinoid activation. Combined with the wide CIs crossing zero and the small magnitude of change observed in CPM, these factors limit our ability to demonstrate a definitive mechanistic pathway. Consequently, the findings regarding pain modulation should be viewed as preliminary, emphasizing that neither intervention consistently triggers the descending inhibitory system in this population.

## 5. Conclusions

While a single session of low‐intensity isometric exercises with BFR elicited modest acute changes in pain sensitivity, this effect was not significantly superior to that of isometric exercise alone in patients following RC repair. Despite the observed statistical signals, the changes did not reach the threshold for clinically meaningful EIH. Furthermore, although baseline clinical status showed some relationship with these effects, the observed correlations were weak, suggesting a limited role for these predictors. These findings should be interpreted cautiously, as unmeasured psychosocial factors and the lack of consistent mechanistic activation, evidenced by the high variability in CPM, indicate that BFR does not provide a robust additional hypoalgesic advantage in this population.

## Author Contributions

Felipe Ponce‐Fuentes: Conceptualization, data curation, formal analysis, investigation, methodology, project administration, resources, supervision, validation, visualization, writing–original draft, and writing–review and editing; Iván Cuyul‐Vásquez: conceptualization, investigation, methodology, resources, writing–original draft, and writing–review and editing; Joaquín Salazar‐Méndez: methodology, writing–original draft, and writing–review and editing; Enrique Lluch: methodology, supervision, validation, writing–original draft, and writing–review and editing; Luis Suso‐Martí: data curation, formal analysis, methodology, supervision, and validation; Chad Cook: supervision, validation, writing–original draft, writing–review and editing, and writing–review and editing; Filip Struyf: conceptualization, data curation, formal analysis, methodology, supervision, validation, writing–original draft, and writing–review and editing; Joaquín Calatayud: conceptualization, data curation, formal analysis, methodology, supervision, validation, writing–original draft, and writing–review and editing; José Granell Casaña: conceptualization, data curation, formal analysis, methodology, supervision, validation, writing–original draft, and writing–review and editing.

## Funding

No funding was obtained for this study.

## Disclosure

All authors approved the final manuscript.

## Ethics Statement

Patients were provided with written information about the purpose, procedures, and safety issues. Written informed consent was obtained in strict accordance with the Declaration of Helsinki. The Scientific Ethics Committee of the RedSalud Clinic of Chile approved the study (protocol number: P‐5.2025).

## Conflicts of Interest

The authors declare no conflicts of interest.

## Supporting Information

Supporting information 1. Carryover effect analysis for the PPT and CPM between sessions *T*_1_, *T*_2_ y *T*_3_.

## Supporting information


**Supporting Information** Additional supporting information can be found online in the Supporting Information section.

## Data Availability

The datasets used and analyzed during the current study are available from the corresponding author upon reasonable request.
