# Supplementary material for: Exercise‐Induced Hypoalgesia Following Blood Flow Restriction in Rotator Cuff Repair Rehabilitation: A Randomized Crossover Clinical Trial
Source: Transl Sports Med. 2026 Apr 16;2026:4698970. doi: 10.1155/tsm2/4698970 (PMC13087449; doi:10.1155/tsm2/4698970)
Supplement: Supplementary file 1 — Supporting Information Additional supporting information can be found online in the Supporting Information section. [file TSM2-2026-4698970-s001.docx]

**Supplementary material 1.** Carryover effect analysis for the PPT and CPM between sessions.

| **Variable** | ***p*-value** | **F** | ***n*_p_^2^** |
| --- | --- | --- | --- |
| PPT, kg/cm^2^ | | | |
| - Affected deltoid | 0.129 | 2.426 | 0.106 |
| - Unaffected deltoid | 0.556 | 0.358 | 0.017 |
| - Affected upper trapezius | 0.949 | 0.004 | 0.000 |
| - Unaffected upper trapezius | 0.556 | 0.359 | 0.017 |
| CPM, % | | | |
| - Affected deltoid | 0.479 | 0.520 | 0.024 |
| - Unaffected deltoid | 0.984 | 0.000 | 0.000 |
| - Affected upper trapezius | 0.326 | 1.013 | 0.046 |
| - Unaffected upper trapezius | 0.147 | 2.498 | 0.134 |

Abbreviations: PPT, Pain Pressure Threshold; CPM, Conditioned Pain Modulation; *n*_p_^2^, partial eta squared.
